# Establishment and characterization of 18 human colorectal cancer cell lines

**DOI:** 10.1038/s41598-020-63812-z

**Published:** 2020-04-22

**Authors:** Soon-Chan Kim, Hyun-Soo Kim, Jae Hyeon Kim, Nahyun Jeong, Young-Kyoung Shin, Min Jung Kim, Ji Won Park, Seung-Yong Jeong, Ja-Lok Ku

**Affiliations:** 1 Korean Cell Line Bank, Laboratory of Cell Biology, Cancer Research Institute, Seoul National University College of Medicine, Seoul, 03080 Korea; 2 Cancer Research Institute, Seoul National University College of Medicine, Seoul, 03080 Korea; 3 Deparntment of Biomedical Sciences, Seoul National University College of Medicine, Seoul, 03080 Korea; 4 Department of Surgery, Seoul National University College of Medicine, Seoul, 03080 Korea; 5 Division of Colorectal Surgery, Department of Surgery, Seoul National University Hospital, Seoul, 03080 Korea

**Keywords:** Colon cancer, Cell growth

## Abstract

Colorectal cancer (CRC) represents the third most frequently diagnosed malignancy worldwide and is the second most common cause of tumor-associated mortalities in Korea. Due to the disease’s aggressive behavior, the 5-year survival rate for CRC patients remains unpromising. Well-characterized cell lines have been used as a biological model for studying the biology of cancer and developing novel therapeutics. To assist *in vitro* studies, 18 CRC cell lines (SNU-1566, SNU-1983, SNU-2172, SNU-2297, SNU-2303, SNU-2353B, SNU-2359, SNU-2373B, SNU-2407, SNU-2423, SNU-2431, SNU-2465, SNU-2493, SNU-2536C, SNU-2621B, SNU-NCC-61, SNU-NCC-376, and SNU-NCC-377) derived from Korean patients were established and characterized in the present study. General characteristics of each cell line including doubling time, *in vitro* morphology, mutational profiles, and protein expressions of CRC-related genes were described. Whole exome sequencing was performed on each cell line to configure mutational profiles. Single nucleotide variation, frame shift, in-frame deletions and insertions, start codon deletion, and splice stop codon mutation of various genes were found and classified based on their pathogenicity reports. In addition, cell viability was assayed to measure their sensitivities to 24 anti-cancer drugs including anti-metabolites, kinase inhibitors, histone deacetylase inhibitors, alkylating inhibitors, and topoisomerase inhibitors, all widely used for various cancers. On testing, five CRC cell lines showed MSI, of which *MLH1* or *MSH6* gene was mutated. These newly established CRC cell lines can be used to investigate biological characteristics of CRC, particularly for investigating gene alterations associated with CRC.

## Introduction

Colorectal cancer (CRC) represents the third most frequently diagnosed tumor worldwide and is the second most common cause of tumor-associated mortalities in Korea^1,2^. It remains the second most perpetual type of tumor in both genders (men: 12.4%; women: 10.1%), and the number of CRC cases continues to increase. Approximately 25% of CRC cases are diagnosed in stage IV, and recurrence with distance metastasis follows after primary resection in nearly 50% of CRC patients^3^. Due to its aggressive behavior, the 5-year relative survival rate remains disapproving^4^. Neoadjuvent therapy is generally performed before surgical resection as single- or multi-agent chemotherapy to improve prognosis^5^. While roughly 50% of CRC patients respond to customary chemotherapy, the majority develop drug resistance through the course of treatment, and relapse or distance metastasis often follows. In recent years, novel anti-cancer agents that target surface growth factor receptors have been developed as adjuvant therapy to decrease the risk of the cancer recurrence^6^.

Well-characterized cell lines have been used as models for studying the biology of cancer and developing novel therapeutics^7^. However, most of the widely used CRC cell lines were derived from Caucasian and African American populations. Accordingly, inter-heterogeneity from ethnic diversity has been biased toward Western countries. To address this need, we established and characterized 18 novel CRC cell cultures (SNU-1566, SNU-1983, SNU-2172, SNU-2297, SNU-2303, SNU-2353B, SNU-2359, SNU-2373B, SNU-2407, SNU-2423, SNU-2431, SNU-2465, SNU-2493, SNU-2536C, SNU-2621B, SNU-NCC-61, SNU-NCC-376, and SNU-NCC-377) from 18 Korean CRC patients. Characterization includes cellular phenotypes, growth rates, mutations of CRC-driver genes, and sensitivities to 24 anti-colorectal cancer drugs that are approved by the National Cancer Institute. The 24 drugs are categorized as anti-metabolites (TAS-102, Capecitabine, 5-FU), kinase inhibitors (Regorafenib, Apitolisib, MK-5108, AZD2014, Afatinib, Buparlisib, Trametinib), histone deacetylase inhibitors (Belinostat, SAHA), alkylating inhibitors (Oxaliplatin), topoisomerase inhibitors (Irinotecan), growth factor receptor inhibitors (Cetuximab, Bevacizumab), natural compounds (Resveratrol, Curcumin, Baicalein, Genistein), and miscellaneous (Lecouvorin calcium, ICG-001, Olaparib). These newly established 18 cell lines can be used to study the molecular biology of CRC, specifically to investigate genomic alterations related to CRC.

## Materials and Methods

### Establishment of cell lines and cell culture

Eighteen human CRC cell lines (SNU-1566, SNU-1983, SNU-2172, SNU-2297, SNU-2303, SNU-2353B, SNU-2359, SNU-2373B, SNU-2407, SNU-2423, SNU-2431, SNU-2465, SNU-2493, SNU-2536C, SNU-2621B, SNU-NCC-61, SNU-NCC-376 and SNU-NCC-377) were established from pathologically proven colorectal tumor tissues acquired from Korean CRC patients. Each participant was given informed consent before cell line establishment and experiment. The detailed procedure was described previously^8^. These novel 18 CRC cell lines were deposited at Korean Cell Line Bank (Seoul, Korea).

### Mycoplasma test

Mycoplasma contamination test was performed as described previously using e-Myco^TM^ kit (iNtRON Biotechnology, INC., Gyeonggi, Korea)^9^. Samples were arranged in the kit, including positive and negative controls for mycoplasma contamination. Mycoplasma control DNA served as a positive control and sterilized distilled water was used as the negative control. PCR amplification was performed under the following conditions: denaturation, 94 °C; annealing, 58 °C; and extension, 75°C. To confirm the specificity of contamination, PCR products were analyzed with gel electrophoresis using 2% agarose gel. PCR amplification and contaminated products had sizes of 570 bp and 260 bp, respectively.

### Growth properties and morphology *in vitro*

Cell growth rate was measured with same method described previously^9^. For growth properties, cells were seeded into 96-well plates at a density of 2.0 × 10^3^ cells/well and were treated with EZ-cytox (DAEIL Lab, Seoul, Korea), a water-soluble tetrazolium salt solution that could be reduced by succinate-tetrazolium reductase to produce formazan dye. After incubating at 37 °C for 2 h, optical density (OD) was assessed at 450 nm using a Multiskan™ GO Microplate Spectrophotometer (Thermo Fisher Scientific, Waltham, MA, USA). The number of cells was analyzed in triplicate at 24-hour intervals for at least 7 days. The doubling time of the cells was calculated from the growth phase. Growth curve and growth properties were drawn and calculated using GraphPad Prism software with normalized OD values. Cell morphology was assessed using an Axiovert 100 microscope at 100× magnification.

### DNA fingerprinting

DNA fingerprinting analysis was performed as decreased before^10^. Briefly, total DNA was isolated from cell pellet by using QIAamp DNA Mini Kit (Qiagen, Hilden, Germany) according to manufacturer’s protocol. Quantified and diluted gDNA solution was added to reaction mixture consisted of Amp FISTR PCR reaction mix, Taq DNA polymerase, and Amp FISTR identifier primer set (Applied Biosystems, CA, USA). DNA was amplified using a GeneAmp PCR System 9700 (Applied Biosystem) with annealing temperature set to 59°C. Gene Scan-500 Rox standard (0.05 μl) and 9 μl oHi-Di Formamide (Applied Biosystem) were added to 1 μl of PCR product of each cell line and denatured at 95 °C for 2 min. The mixture was then analyzed with a 3500 xL Genetic Analyzer (Applied Biosystems).

### Drug sensitivity test

At density of 2 × 10^5^ cells/well, tumor cells were seeded into a 96-well plate. Optimal concentrations of anti-cancer drugs were then used to treat 18 CRCs. These concentrations were: 100 μg/ml of TAS-102, 100 μg/ml of Regorafenib, 1000 μg/ml of Leucovorin calcium, 1000 μg/ml of Capecitabine, 50 μg/ml of Apitolisib, 100 μg/ml of Belinostat, 50 μg/ml of Trametinib, 50 μg/ml of Cyclopamine, 100 μg/ml of ICG-001, 100 μg/ml of Buparlisib, 50 μg/ml of SAHA, 50 μg/ml of Afatinib, 5 μg/ml of AZD2014, 100 μg/ml of MK-5108, 50 μg/ml of Olaparib, 100 μg/ml of Irinotecan, 50000 μg/ml of 5-FU, 100 μg/ml of Oxaliplatin, 100 μg/ml of Baicalein, 100 μg/ml of Curcumin, 100 μg/ml of Genistein, 200 μg/ml of Resveratrol, 1000 μg/ml of Cetuximab, and 1000 μg/ml of Bevacizumab. The 96-well plate containing anti-cancer drugs was incubated for 72 h at 37 °C. After incubation, 10 ul EZ-Cytox solution was applied to each well. After the plate was incubated for 2 h at 37 °C, optical density value was assessed at 450 nm with a Multiskan™ GO Microplate Spectrophotometer (Thermo Fisher Scientific).

### Western blotting analysis

Detailed procedure was described previously^9^. Cells were harvested with a cell scraper after washing with cold PBS. Whole protein was extracted with EzRIPA buffer (ATTO Co., Tokyo, JAPAN) supplied with 1% protease inhibitor and 1% phosphatase inhibitor in accordance with the cell viability assay time frame. The volume of lysis buffer was adjusted to the number of cells collected in each vial. The protein concentration was determined by SMART^TM^ micro BCA protein assay kit (Intron biotechnology, Gyeonggi, Korea). Proteins in equal amounts were loaded on a 4-12% Bis-Tris gel (Invitrogen) and run at 50 volts for 2 h. Proteins on gel were then transferred to a PVDF membrane (Invitrogen) by electro-blotting with constant current of 80 mA at 4 °C overnight. Proteins on transferred membrane were blocked by incubating with 1.5% to 2.0% skim milk in 0.05% Tween 20-TBS buffer including 1 mM MgCl_2_ at room temperature for an hour. The membrane was then incubated with primary antibodies against EGFR (abcam, Cambridge, United Kingdom) (1:2000), HER2 (abcam, Cambridge, United Kingdom) (1:1000), MLH1 (Santa Cruz Biotechnology, TX, USA) (1:500), MSH2 (Santa Cruz Biotechnology, TX, USA) (1:500), EpCAM (Santa Cruz Biotechnology, TX, USA) (1:1000), E-cadherin (abcam, Cambridge, United Kingdom) (1:1000), vimentin (abcam, Cambridge, United Kingdom) (1:2000), and β-actin (Santa Cruz Biotechnology, TX, USA) (1:100) followed by incubation with mouse or rabbit IgG 2^nd^ antibody (Jackson Immunoresearch, PA, USA) (1:5000) conjugated with peroxidase that matched with the primary antibody used. Chemiluminescent working solution WESTZOL^TM^ (Intron biotechnology) was then used to treat the membrane which was then exposed to Fuji RX film (Fujifilm, Tokyo, Japan) for 1-5 minutes.

### Whole exome sequencing

Detailed procedure was described previously^9^. SureSelect sequencing libraries were prepared using SureSelect Human All Exon 50 Mb Kit (Agilent) according to manufacturer’s instructions using a Bravo automated liquid handler. Three micrograms of genomic DNA were fragmented to a median size of 150 bp using a Covaris-S2 instrument (Covaris, MA, USA). Adapter ligated DNA was amplified by PCR. PCR product quality was then assessed by capillary electrophoresis. Hybridization buffer and DNA blocker mix were incubated at 95 °C for 5 minutes and 65 °C for 10 min in a thermal cycler. The hybridization mixture was then added to a bead suspension and incubated at RT for 30 min while mixing. These beads were washed and DNA was eluted from beads with 50 ml SureSelect elution buffer (Agilent). The flow cell was then loaded on a HiSeq. 2500 sequencing system (Illumina).

### MSI test

Detailed procedure was described previously^11^. For microsatellite instability (MSI) analysis, BAT25 and BAT26 (two mononucleotide microsatellite markers) were evaluated using a capillary-based sequencing analysis^8^. PCR was performed as described above except that forward primers were labeled with a fluorescent dye. Labeled samples were run on an ABI 3730 genetic analyzer (Applied Biosystems). GeneMapper software v4.0 (Applied Biosystems) was used to calculate the size of each fluorescent PCR product. For gel-based MSI analysis, desired fragments were amplified in the presence of [a-P32] deoxycytidine triphosphate. PCR products were denatured and separated on 6 M urea/7%polyacrylamide gels run at 60 W.

### Ethics approval and consent to participate

The study protocol was approved by the Institutional Review Board of Seoul National University Hospital (IRB No. H-1102-098-357). The study was performed in accordance with the Declaration of Helsinki.

## Results

### General characteristics of CRC cell lines

Human specimens were obtained from CRC patients who underwent surgeries at Seoul National University (SNU) Hospital from 1999 to 2008. Eighteen colorectal carcinoma cell lines (SNU-1566, SNU-1983, SNU-2172, SNU-2297, SNU-2303, SNU-2353B, SNU-2359, SNU-2373B, SNU-2407, SNU-2423, SNU-2431, SNU-2465, SNU-2493, SNU-2536C, SNU-2621B, SNU-NCC-61, SNU-NCC-376, and SNU-NCC-377) were established in RPMI 1640 medium supplemented with 10% FBS. *In vitro* and *in vivo* characteristics of newly established 18 CRC cell lines are summarized in Tables 1 and 2. All cell lines were free of contamination by mycoplasma (data not shown).

**Table 1:** *In vitro* characteristics of newly established 18 CRC cell lines.

Cell Line	Primary tumor site/culture site	Date of initiation	Doubling time (day)	Growth pattern
SNU-1566	Colon/ Primary	1999-01-04	2.856	Adherent
SNU-1983	Colon/ Primary	2002-05-06	1.474	Adherent
SNU-2172	Colon/ Primary	2004-01-15	2.122	Adherent
SNU-2297	Colon/ Primary	2006-06-22	1.721	Adherent
SNU-2303	Colon/ Primary	2006-08-17	1.982	Adherent
SNU-2353B	Colon/ Lymph node	2007-08-02	3.157	Adherent
SNU-2359	Colon/ Primary	2007-08-23	2.937	Floating
SNU-2373B	Colon/ Liver	2007-11-29	2.335	Adherent
SNU-2407	Rectal/ Primary	2008-07-03	3.031	Adherent
SNU-2423	Colon/ Serosal	2008-10-26	2.296	Adherent
SNU-2431	Colon/ Primary	2009-04-09	5.759	Adherent
SNU-2465	Colon/ Primary	2010-03-05	2.133	Adherent
SNU-2493	Colon/ Primary	2010-08-05	2.113	Floating
SNU-2536C	Colon/ Liver	2010-11-18	3.305	Adherent
SNU-2621B	Colon/ Ascites	2012-05-07	1.346	Adherent
SNU-NCC-61	Colon/ Primary	2002-11-22	3.283	Adherent
SNU-NCC-376	Colon/ Primary	2005-10-11	3.147	Adherent
SNU-NCC-377	Colon/ Primary	2005-10-13	2.449	Adherent

**Table 2:** *In vivo* characteristics of newly established 18 CRC cell lines.

Cell Line	Age	Sex	Family History	Tumor Size (cm)	T	N	M	Pre. Op chemo regimen	Pre. Op radio regimen	Recurrence-free survial
SNU-1566	38	M	First degree and second degree (CRC) (HNPCC)	n/a	n/a	n/a	n/a	None	n/a	n/a
SNU-1983	50	M	None	6.5	3	0	0	5-FU	None	206
SNU-2172	69	M	Bile Duct Cancer (Mother)	8	4	2	1	None	None	131
SNU-2297	55	M	Breast Cancer (Mother/Sister)	4.5	4	2	1	FOLFOX	None	727
SNU-2303	30	M	First degree and second degree (CRC)	7.5	3	0	0	5-FU	None	2210
SNU-2353B	63	M	Pancreatic Cancer (Father)	6.5	4	2	1	None	None	2295
SNU-2359	73	M	None	9	4	2	1	None	None	29
SNU-2373B	74	M	None	6	3	1	1	XELOX	None	2178
SNU-2407	76	M	Breast Cancer (Sister), Second degree(CRC)	10	4	0	0	XELOX	None	1842
SNU-2423	57	F	First degree and second degree(CRC) (HNPCC)	n/a	n/a	n/a	n/a	n/a	n/a	n/a
SNU-2431	n/a	M	None	n/a	n/a	n/a	n/a	n/a	n/a	n/a
SNU-2465	66	F	None	2.3	4	2	1	FOLFIRI	None	339
SNU-2493	58	F	None	7	4	0	0	FOLFOX	None	1864
SNU-2536C	69	M	None	3	3	2	1	FOLFOX	None	1197
SNU-2621B	51	M	n/a	n/a	n/a	n/a	n/a	n/a	n/a	n/a
SNU-NCC-61	49	M	n/a	n/a	n/a	n/a	n/a	n/a	n/a	n/a
SNU-NCC-376	73	M	n/a	n/a	n/a	n/a	n/a	n/a	n/a	n/a
SNU-NCC-377	64	M	n/a	n/a	n/a	n/a	n/a	n/a	n/a	n/a

### Morphology and growth properties of CRC cell lines

**Figure 1:**
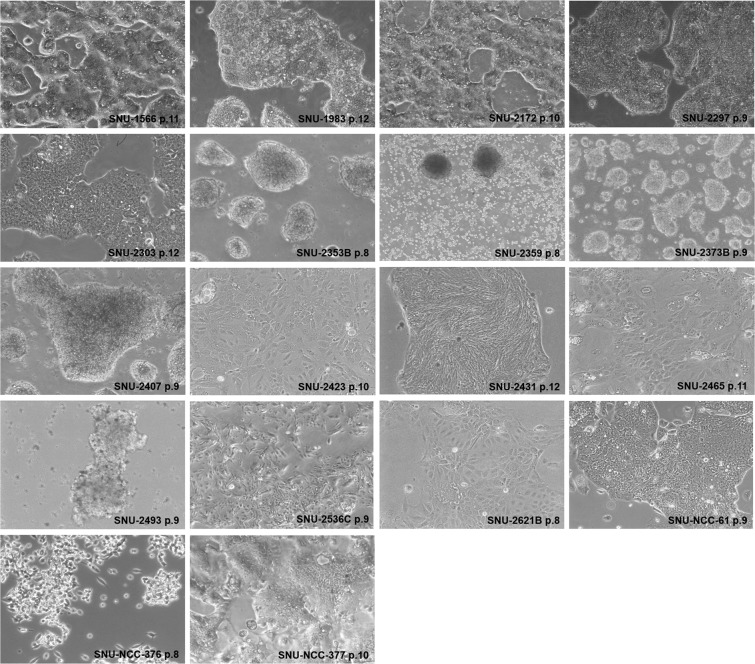
*in vitro* morphology of CRC cell lines. Cell images were acquired at 100× magnification. The majority of tumor cells displayed a polygonal shape and had exhibited round-to-oval nuclei with prominent single-to-double nucleoli.

Cell images were acquired using Axiovert 100 microscope at 100× magnification (Fig. 1). On *in vitro* cultivation, sixteen CRC cell lines (SNU-1566, SNU-1983, SNU-2172, SNU-2297, SNU-2303, SNU-2353B, SNU-2373B, SNU-2407, SNU-2423, SNU-2431, SNU-2465, SNU-2536C, SNU-2621B, SNU-NCC-61, and SNU-NCC-377) grew as monolayers of substrate-adherent cells. SNU-NCC-61cell line showed spindle morphology while other cell lines showed polygonal morphology. SNU-2359 and SNU-2493 grew as floating clumps. SNU-NCC-376 cell line formed floating and adherent aggregates (Fig. 1). The majority of tumor cells displayed a polygonal shape and had exhibited round-to-oval nuclei with prominent single-to-double nucleoli. Each cell line was passaged at least three times prior to characteristic analysis. Population doubling times ranged from 32 to 138 hours.

### DNA fingerprinting of 18 CRC cell lines

Fifteen tetranucleotide repeat loci and the gender-determining marker amelogen were heterogeneously distributed in each cell line, without cross-contamination (Table 3). They were also matched with the STR profiles of cell lines with passage 0 or 1 (including original tissue mass) in order to confirm that the established cell lines were not cross-contaminated with other patient material (Supplementary Table 1).

**Table 3:** DNA fingerprinting analysis using 16 STR loci for 18 CRC cell lines.

Cell Line	D8S1179	D21S11	D7S820	CSF1PO	D3S1358	TH01	D13S317	D16S539
SNU-1566	12,14	31,32	8,11	9,10	14,16	9	10,13	9,12
SNU-1983	12,17	28,35.2	12,13	10,12	15,17	9	8,11	10,12
SNU-2172	12,13	30,32.2	10	8,12	16	10	8,10	10,11
SNU-2297	13,14	29	8,9	10	15	6,9	8,10	12,13
SNU-2303	11,13	29,35.2	11,12	9,10,11	15,16	9	8,11	11,12
SNU-2353B	13	29	10,11	11	15,17	9	8,12	11,13
SNU-2359	13,14	28.2,30	12	10	14,15	7	8	10,12
SNU-2373B	12,14	31.2	11,12	12	15,17	9	9,12	9,14
SNU-2407	12,13	30,31	11	12	15,18	7,10	9	9,12
SNU-2423	10,16	28,30	11	10,13	14,17	8,9	8	10,12
SNU-2431	12,15	31	10	11,13	15,17	9	9,11	9
SNU-2465	11,12	29,31.2	9,12	9,12	15	8,9	8,11	9
SNU-2493	10,13	32.2	11	12	15,16	9	9	11
SNU-2536C	15	29,30	8	10,11	16	6,9	8,11	11
SNU-2621B	14	28,32	11,12	10,11	15,19	7,9	9,10	9,12
SNU-NCC-61	11,15	30	10,12	10,12	15	6,9	12	11
SNU-NCC-376	13	31,32.2	13	11,13	15	7	12	9
SNU-NCC-377	12,13	32.2	10,12	13	15	6	8,9	10,11
**Cell Line**	**D2S1338**	**D19S433**	**Vwa**	**TPOX**	**D18S51**	**Amelogenin**	**D5S818**	**FGA**
SNU-1566	16,24	12,16	17,18	7,8	14,16	X,Y	12	22,4
SNU-1983	20,23	12,14	20,22	8,10	12,23	X,Y	10,13	20,22
SNU-2172	19,23	13,14	17	8	13	X,Y	12	21
SNU-2297	23,24	13	17,20	8	13	X, Y	12	25
SNU-2303	18,19	12,13	17,18	8,10	11,13	X,Y	10,11	22,25
SNU-2353B	20	13,14.2	17	11	15,16	X,Y	10,13	22
SNU-2359	23	13	16,19	11	14,15	X,Y	10	21,22
SNU-2373B	22	13,14	17	8,9	16	X,Y	14	19,21
SNU-2407	22,23	13,16.2	18,19	11	16	X	11	24
SNU-2423	17,19	12,14.2	16	9	10,21	X	10,14	21,24
SNU-2431	17,20	13.2,15	16,18	11	15	X, Y	10,11	20,21
SNU-2465	17,25	13,14	16	8	17	X	10,15	23,26
SNU-2493	23	13.2,14	14,16	11	18	X	9,11	26
SNU-2536C	18,23	13,14.2	16,18	8,10	14	X, Y	10,13	22
SNU-2621B	21,25	13,14	15,22	7,9	15,19	X,Y	8,9	20,23
SNU-NCC-61	18,19	14.2,15	19	8,9	17	X, Y	11,13	22,23
SNU-NCC-376	17,24	13	16	8,11	14,17	X,Y	10,13	19,21
SNU-NCC-377	17,20	13	16,17	8,11	17	X, Y	12,16	24

### Expression levels of growth factor receptor and EMT proteins in 18 CRC cell lines

**Figure 2:**
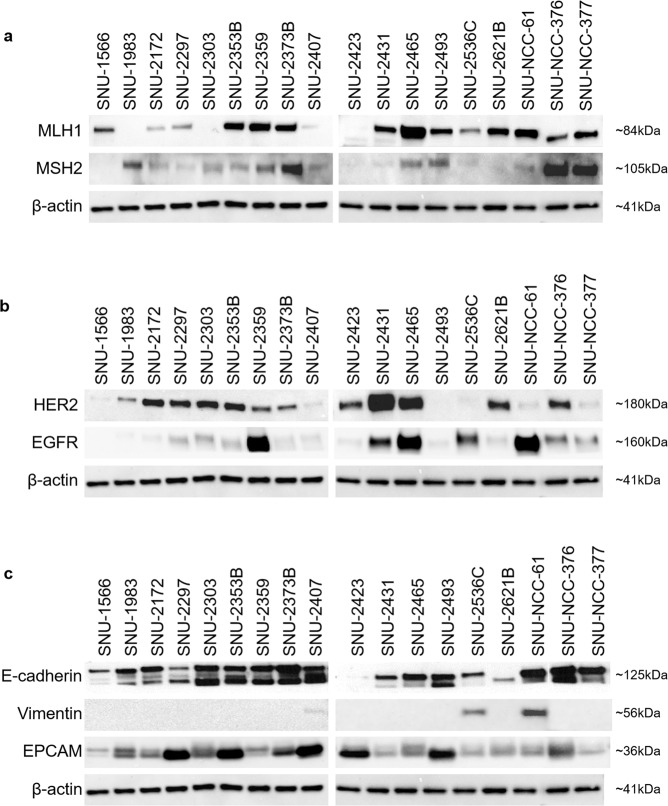
The expression level of the mismatch repair protein. (**a**) MLH1 and MSH2, in 18 CRC cell lines. The protein expression level was detected by western blotting assay. The expression level of growth factor receptor and EMT proteins. (**b**) The expression level of growth factor receptor, HER2 and EGFR was assessed by western blot analysis. (**c**) The expression level of the EMT proteins, EPCAM, E-cadherin and Vimentin was assessed by western blot analysis.

Protein expressions of *MLH1* and *MSH2* of newly established cell lines were analyzed in accordance with their mutational profiles. Three cell lines (SNU-1983, SNU-2434 and SNU-3030) had pathogenic mutations in *MLH1* and the protein expression was exclusively low accordingly. Two cell lines (SNU-2359 and SNU-2493) harbored benign mutation in *MLH1* (c.655 A > G/p.Ile219Va), and protein structure was not affected. Although no pathogenic *MSH2* mutation was present in the newly established CRC cell lines, the protein expression of *MSH2* was varying, which implicated the protein expression of *MSH2* was determined by RNA splicing or epigenetical alternations (Fig. 2a). Four cell lines (SNU-2359, SNU-2431, SNU-2465 and SNU-NCC-61) exhibited augmented *EGFR* level. SNU-2431 and SNU-2465 had increased expression of both *EGFR* and *HER2* (Fig. 2b). Expression levels of EMT-related proteins, E-cadherin, EPCAM and vimentin were analyzed according to the *in vitro* molphology (Fig. 2c). E-cadherin was significantly decreased in SNU-2423, while EPCAM was expressed in all cell lines. Vimentin was exclusively expressed in SNU-2536C and SNU-NCC-61. Both cell lines grew as monolayers of substrate-adherent cells with adherent aggregates.

### Genomic analysis

**Figure 3:**
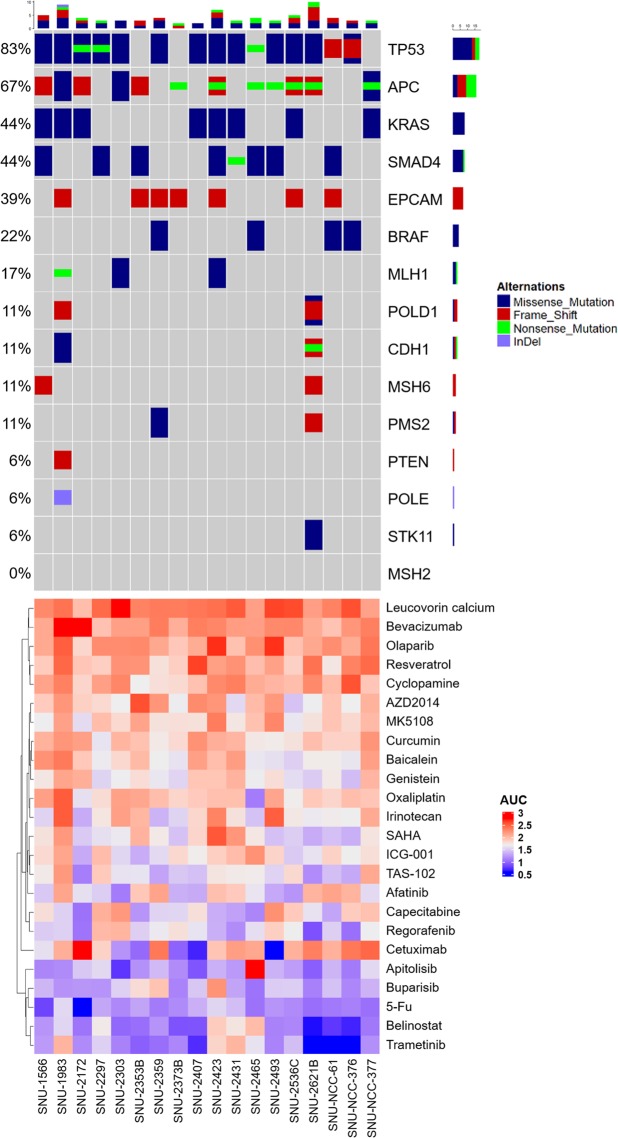
Heat map pattern of AUC for anticancer drug response of 18 CRC cell lines with the mutational landscape. Blue color indicates the better response to anticancer drug and red color indicate the worse response to anticancer drug 15 genes associated with colorectal cancer were analyzed.

Fifteen genes in developing CRC were screened in the 18 newly established CRC cell lines. Using Clinvar database (www.ncbi.nlm.nih.gov/clinvar), we determined pathogenic mutations. Results are summarized in Fig. 3, Table 4 and Supplementary Table 2. Mutations included in the Fig. 3 are only pathogenic mutations indicated by Clinvar database. Supplementary Table 2 includes the entire mutations in which their clinical meanings were in question. The most common actionable alterations across the sample sets were *TP53* (83%) and *APC* (67%). *KRAS* and *SMAD4* mutations were also prevalent in the sample sets at 44%. The most hyper-mutated cell line was SNU-2621B (10 mutations). Genes that are related to DNA repair such as *POLD1*, *MSH6*, and *PMS2* were mutated in the SNU-2621B cell line. Similarly, SNU-1983 was also hyper-mutated (9 mutations) and DNA repair genes such as *MLH1* and *POLD1* were mutated. The truncation mutations of *MLH1* and *MSH6* genes in SNU-1566, SNU-1983 and SNU-2621B cell lines were confirmed with Sanger sequencing (Table 4, Supplementary Figs. 1–3).

**Table 4:** Abnormalities of the *hMLH1, hMSH2, hMSH6* genes.

Cell line	Abnormalities of the *hMLH1,hMSH2,hMSH6* genes				
	MSS/MSI	Gene	Codon	Necleotide change	a.a change
SNU-1566	MSI	*MSH6*	3261	3261delC	p.Phe1088fs
SNU-1983	MSI	*MLH1*	1997	1997G > A	p.Trp666*
SNU-2172	MSS				
SNU-2297	MSS				
SNU-2303	MSI	*MLH1*	1721	1721T > C	p.Leu574Pro
SNU-2353B	MSS				
SNU-2359	MSS				
SNU-2373B	MSS				
SNU-2407	MSS				
SNU-2423	MSI	*MLH1*	1721	1721T > C	p.Leu574Pro
SNU-2431	MSS				
SNU-2465	MSS				
SNU-2493	MSS				
SNU-2536C	MSS				
SNU-2621B	MSI	*MSH6*	3261	3261delC	p.Phe1088fs
SNU-NCC-61	MSS				
SNU-NCC-376	MSS				
SNU-NCC-377	MSS				

### Anticancer drug response of 18 CRC cell lines

Areas under curve (AUCs) of 18 CRC cell lines in response to NIH approved 24 anti-cancer drugs, including anti-metabolite (TAS-102, Capecitabine, 5-FU), kinase inhibitor (Regorafenib, Apitolisib, MK-5108, AZD2014, Afatinib, Buparlisib, Trametinib), histone deacetylase inhibitor (Belinostat, SAHA), alkylating inhibitor (Oxaliplatin), topoisomerase inhibitor (Irinotecan), growth factor receptor inhibitor (Cetuximab, Bevacizumab), natural compounds (Resveratrol, Curcumin, Baicalein, Genistein), and miscellaneous (Lecouvorin calcium, ICG-001, Olaparib), were estimated (Fig. 3). CRC cell lines were uniformly sensitive to Apitolisib, Trametinib, Belinostat, 5-FU, and Buparlisib with exceptions of SNU-2423 and SNU-2465. and resistant to Cetuximab, Bevacizumab, Leucovorin calcium, Olapraib, cyclopamine, and Resveratrol.

## Discussion

New CRC cases continue to increase. At the time of detection, many CRC cases have already progressed to stage IV, which makes surgical resection unfeasible, and nearly 50% of CRC cases have shown recurrence or distance metastasis after primary resection^12^. Although there has been much research on inventing novel therapeutics, the molecular basis of drug response and aggressive behavior remains obscure due to its genetic intricacy, and more comprehensive analysis is called for to refine regimes for treatment and prevention^13^.

The importance of human CRC cell lines lies in their similarity to original tissues and their renewability, which facilitate the study of human CRC. Several CRC cell lines such as HCT‐116, LoVo, SW‐480, and WiDr have accelerated the CRC research. Nevertheless, those accessible CRC cell lines are somewhat obsolete and possibly acquire genetic alternations as passaging^14^. Clinical correlation between original human materials and cancer cell lines can decrease due to the accumulation of genetic aberrations with increasing subculture numbers^15–19^. Therefore, novel CRC cell lines can deliver suitable biological models for investigating a broader spectrum of molecular characteristics of CRC. J. H. Oh *et al*. established 12 human CRC cell lines from 6 primary and 6 metastatic tumors of 11 Korean CRC patients^10^. In addition, J. L. Ku *et al*. established 13 CRC cell lines from 10 primary and 3 metastatic tumors of 13 Korean patients^8^. In this study, we established 18 novel CRC cell lines from 13 primary and 5 metastatic tumors of Korean patients who underwent surgical resection from 1999 to 2008 in SNU Hospital. Novel cell lines established through this study will be deposited to the Korean Cell Line Bank at various passages.

Nearly 15% of sporadic CRC cases show the MSI phenotype, which is prompted by inactivation of mismatch repair (MMR) genes such as *MLH1*, *MSH2*, and *MSH6*^20^. Hereditary non-polyposis CRC, which accounts for 2–5% of all CRC cases is also concurrent with germline mutations in MMR genes. Nearly 90% of reported mutations in MMR genes were harbored in *MLH1* and *MSH2*^21,22^. In this study, five cell lines harbored pathogenic mutations in MMR genes. *MLH1* was mutated in SNU-1983, SNU-2359, SNU-2434, SNU-2493, and SNU-3030. Among these cell lines, three (SNU-1983, SNU-3030, and SNU-2434) had pathogenic mutations in *MLH1*, and the protein expression was exclusively low accordingly. Interestingly, we found no pathogenic *MSH2* mutation in the newly established CRC cell lines. Although Wei *et al*. reported that there were different patterns of *MSH2* and *MLH1* mutations between Asian and Caucasian population^23^, the prevalence of *MLH1* mutation in comparison with *MSH2* mutation in an Asian population has not been reported. Although we found no pathogenic *MSH2* mutation, the protein expression of *MSH2* varied, which implied that the protein expression of *MSH2* was determined by RNA splicing or epigenetic alternations. Two (SNU-1566, SNU-2423) of these five cell lines were derived from patients with hereditary non-polyposis CRC.

*APC, KRAS*, and *tp53* are frequently abberant genes in CRCs^15^, and these three genes were mostly mutated in the CRC cell lines characterized in this study as well. Most of the identified *APC* germline alternations are nonsense mutations or frameshift mutations near the 5’ end of the gene, which truncated the protein structure^24^. We considered *APC* mutations pathogenic when they were reported in Clinvar (https://www.ncbi.nlm.nih.gov/clinvar), and the types of pathogenic *APC* mutations we identified in this study were also nonsense or frameshift.

*KRAS* serves as a fundamental mediator in the transduction of several growth or differentiation factor stimuli^25^. Most aberrations in *KRAS* harbor codons 12, 13, 59, and 61^26^. In this study, *KRAS* mutations gene were harbored in 8 of 18 cell lines (44%). Two cell lines (SNU-1566 and SNU-2423) had a mutation at codon 13, and six lines had a mutation at codon 12. Mutation types were G to A or G to T transitions.

Nearly 50% of CRC cases have several genetic alternations in *tp53*^27^. In this study, mutations of *tp53* were present in 16 (88%) of the 18 cell lines. All *tp53* mutations were at codons 72 (n = 12), 74 (n = 1), 176 (n = 1), 196 (n = 1), 213 (n = 1), 245 (n = 1), 337 (n = 1), 342 (n = 1), 800 (n = 1), and c.376-1 G > A in our study. Interestingly, pPro72Arg mutation is commonly found in gastric cancer^28^. This mutated codon is associated with colorectal cancer^29^. *SMAD4* serves as the fundamental component of TGF-β signaling, and it is reported to be inactivated in many types of tumor including pancreas, stomach, and colon^30^. *SMAD4* mutation has been found in 10~35% of CRC^31–34^. Similarly, we found mutation of *SMAD4* in 2 (10.5%) of 18 cell lines. *SMAD4* mutations were at codons 386 (n = 1) and 442 (n = 1). *PTEN* mutations are known to occur in 5–14% of CRC^35–37^. *PTEN* serves as an anti-oncogene. Over-activation of PI3K/AKT pathway is mainly associated with loss of *PTEN*^38^. In this study, we found mutation of *PTEN* in only 1 of the 18 lines (5.2%), SNU-1983. SNU-1983 had *KRAS* mutation without *BRAF* or *PIK3CA* mutation. Mutations in *BRAF*, specifically valine-to-glutamate change at residue 600 (V600E), account for approximately 10% of CRC cases^39^. The present study showed *BRAF* mutation with *V600E* in 4 (21%) of 18 lines. *STK11* regulates cell polarity and is a tumor suppressor. This gene is mainly related to Peutz-Jeghers syndrome^40^. Cadherin-1 (*CDH1*), in the classical cadherin superfamily, is associated with cancer proliferation and invasiveness^41^. SNU-2621B had mutation in *STK11* and *CDH1*, but no other cell lines had these mutations. SNU-2621B had one *STK11* mutation (pGly163) and two *CDH1* mutations (pArg74* and Arg800fs). Two *CDH1* mutations (pArg74* and Arg800fs) usually occur in gastric cancer^42^.

All 18 CRC cell lines were sensitive to Apitolisib, Trametinib, Belinostat, 5-FU, and Buparlisib. Interestingly, SNU-2465 was resistant to Apitolisib, whereas all other lines were susceptible. All 18 CRC cell lines were resistant to Cetuximab, Bevacizumab, Leucovorin calcium, Olapraib, Cyclopamine, and Resveratrol. These novel cell lines will be deposited at the Korean Cell Line Bank and distributed worldwide for those who study colorectal cancer. These lines can be used as valuable materials to investigate biological properties of heterogeneous CRC.

## Supplementary information

Supplementary Information.
