# Supplementary material for: Establishment and characterization of 18 human colorectal cancer cell lines
Source: Sci Rep. 2020 Apr 22;10:6801. doi: 10.1038/s41598-020-63812-z (PMC7176734; doi:10.1038/s41598-020-63812-z)
Supplement: Supplementary file 1 — Supplementary Information. [file 41598_2020_63812_MOESM1_ESM.pdf]

Establishment and characterization of 18 human colorectal cancer cell lines

Soon-Chan Kim[1,2,3 †], Hyun-Soo Kim[1,2 †], Jae Hyeon Kim[1,2], Nahyun Jeong[1,2], Young-Kyoung Shin[1,2], Min Jung Kim[2,4,5], Ji Won Park[2,4,5], Seung-Yong Jeong[2,4,5], and Ja-Lok Ku[1,2,3]

[1]Korean Cell Line Bank, Laboratory of Cell Biology, Cancer Research Institute, Seoul National University College of Medicine, Seoul 03080, Korea

[2]Cancer research Institute, Seoul National University College of Medicine, Seoul 03080, Korea

[3]Deparntment of Biomedical Sciences, Seoul National University College of Medicine, Seoul 03080, Korea

[4]Department of Surgery, Seoul National University College of Medicine, Seoul 03080, Korea

[5]Division of Colorectal Surgery, Department of Surgery, Seoul National University Hospital, Seoul 03080, Korea

† Soon-Chan Kim and Hyun-Soo Kim contributed equally to this work

Running title: Characterization of human colorectal cancer cell lines.

* *Correspondence to*:

Ja-Lok Ku

Laboratory of Cell Biology, Cancer Research Institute, Seoul National University College of Medicine, 103, Daehak-ro, Jongno-gu, Seoul 03080, Korea. Fax: +82-2) 742-0020.

E-mail: kujalok@snu.ac.kr

Seung-Yong Jeong, Department of Surgery, Seoul National University College of Medicine, 103, Daehak-ro, Jongno-gu, Seoul 03080, Korea

E-mail: syjeong@snu.ac.kr

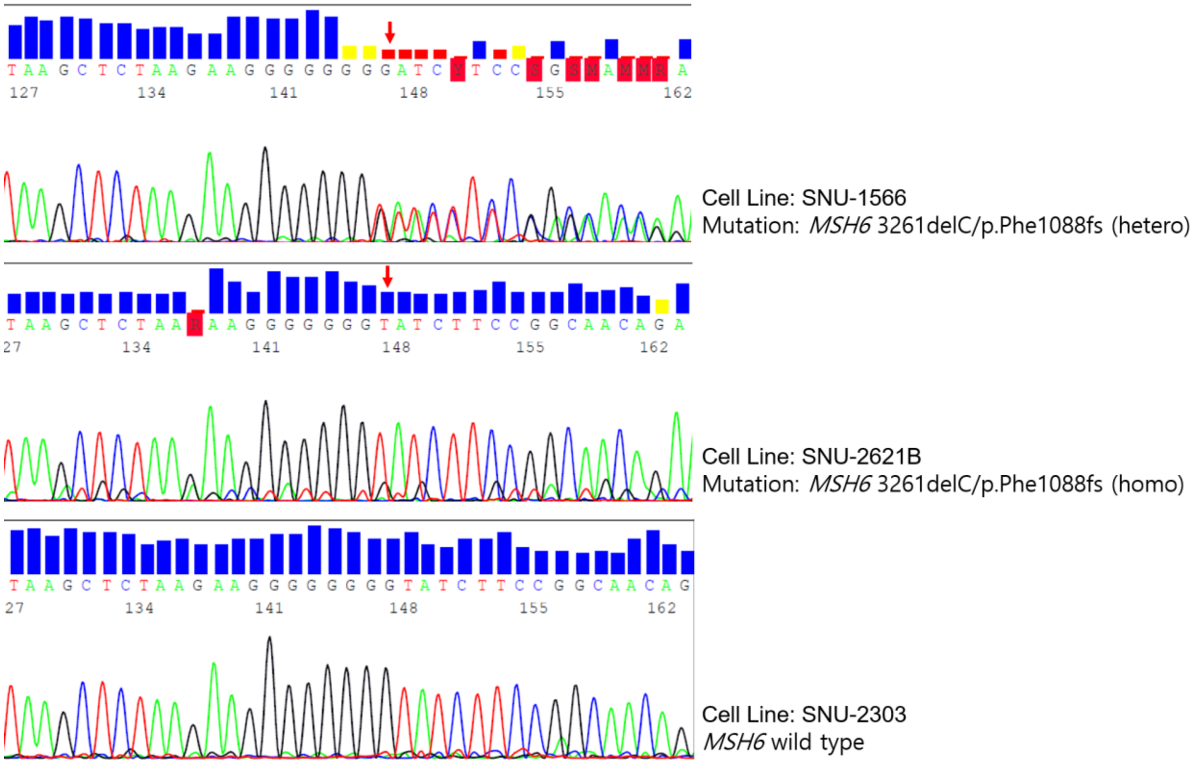

Supplementary figure 1. Sanger sequencing confirmation of MSH6 3261delC/p.Phe1088fs mutations. MSH6 3261delC/p.Phe1088fs mutation in SNU-2303 and SNU-2423 cell lines which was detected in silico analysis was re-confirmed by sanger sequencing.

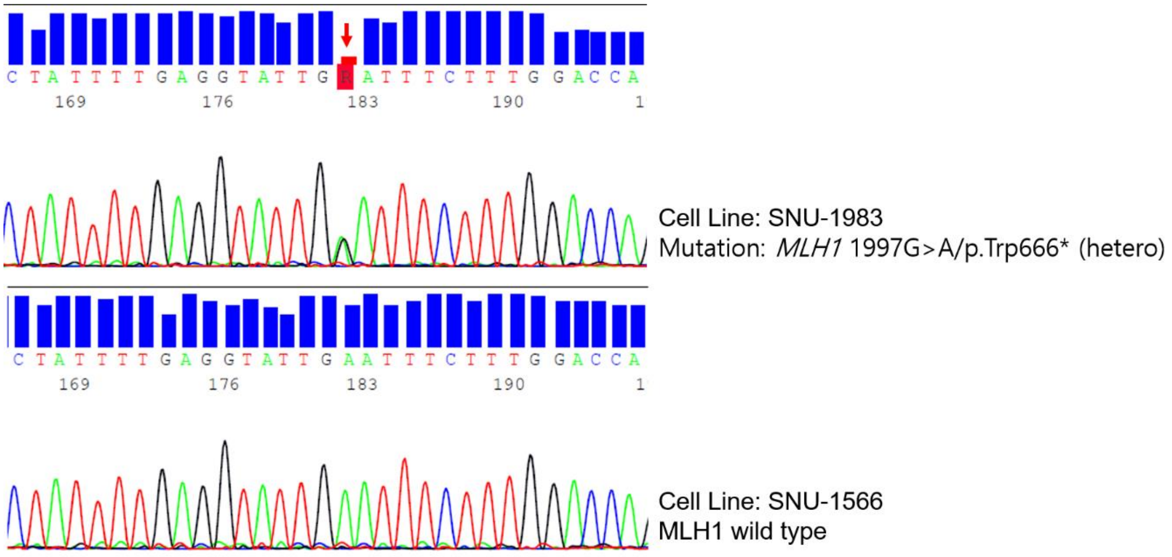

Supplementary figure 2. Sanger sequencing confirmation of MLH1 1997G>A/p.Trp666* mutations. MLH1 1997G>A/p.Trp666* mutations in SNU-1983 cell line which was detected in silico analysis was re-confirmed by sanger sequencing.

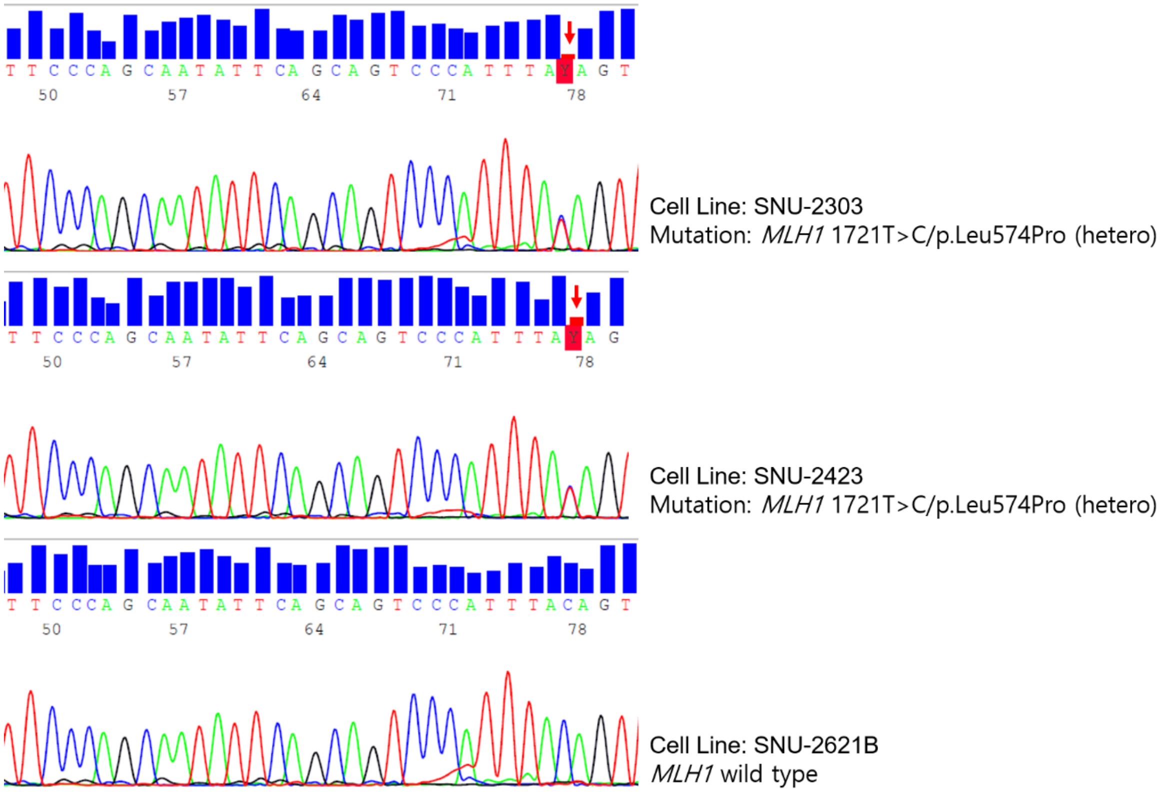

Supplementary figure 3. Sanger sequencing confirmation of MLH1 1721T>C/p.Leu574Pro mutations. MLH1 1997G>A/p.Trp666* mutations in SNU-2303 and 2423 cell lines which was detected in silico analysis was re-confirmed by sanger sequencing.

Supplementary Table 1. STR profiles of initial passages of 18 CRC cell lines

Cell Line	D8S1179	D21S11	D7S820	CSF1PO	D3S1358	TH01	D13S317	D16S539
SNU-1566	12,14	31,32	8,11	9,10	14,16	9	10,13	9,12
SNU-1983	12,17	28,35.2	12	10,12	15,17	9	8,11	10,11
SNU-2172	12,13	30,32.2	10	8,12	16	10	8,10	10,11
SNU-2297	13,14	29	8,9	10	15	6,9	8,10	12,13
SNU-2303	11,14	29,30,33.2,34.2	11,13	9,10,11	15	9	8,10,11	11,12
SNU-2353B	13	29	10, 11	11	15,17	9	8,12	11,13
SNU-2359	13,14	28.2,30	12	10	14,15	7,9	8,9	10,12
SNU-2373B	12,14	30,31.2	11,12	12	15,17	9	9,12	9,14
SNU-2407	12,13	30,31	11	12	15,18	7,10	9	9,12
SNU-2423	10,11,15,16	29,31	11,12	9,10,11,13	14,15,16,17	9	8	10,12
SNU-2431	12,15	31	10	11,13	15,17	9	9,11	9,10
SNU-2465	11,12	29,31.2	9,12	9,12	15	8,9	8,11	9
SNU-2493	10,13	29,32.2	11	12	15,16	7,9	9	11
SNU-2536C	15	29,30	8,11	10,11	16	6,9	8,11	11
SNU-2621B	14	28,32	11,12	10,11	15,19	7,9	9,10	9,12
SNU-NCC-61	11,15	30	10,12	10,12	15	6,9	8,12	11
SNU-NCC-376	13	31,32.2	13	11,13	15	7	12	9
SNU-NCC-377	12,13	32.2	10,12	13	15	6	8,9	10,11

Continued

Cell Line	D2S1338	D19S433	Vwa	TPOX	D18S51	Amelogenin	D5S818	FGA
SNU-1566	16,24	12,16	17,18	7,8	14,16	X,Y	12	22,24
SNU-1983	20,22	12,14	20,22	8,10	12	X,Y	10,13	20,22
SNU-2172	19,23	13,14	17	8	13	X,Y	12	21
SNU-2297	23,24	13	17,20	8	13	X	12	25
SNU-2303	18,19	12,13,14	17,18	8,10,11	10,11,13	X,Y	10,11	22,23,24
SNU-2353B	20,23	13,14.2	17,18	11	15,16	X,Y	10,13	20,22
SNU-2359	23	13	16,19	11	14,15	X,Y	10,12	21,22
SNU-2373B	22	13	17	8,9	16	X,Y	14	19,21
SNU-2407	22,23	13,16.2	18,19	11	16	X	11	24
SNU-2423	17,19	12,14.2	16	9	13,14,20,21	X	9,12,14	20,21,22,23
SNU-2431	17,20	13.2,15	16,18	11	15	X,Y	10,11	20,21
SNU-2465	17,25	13,14	16	8	17	X	10,15	23,26
SNU-2493	23,24	13.2,14	14,16	11	13,18	X	9,11	25,26
SNU-2536C	18,23	13,14.2	16,18	8,10	13,14	X,Y	10,13	22
SNU-2621B	21,25	13,14	15,22	7,9	15,19	X,Y	8,9	20,23
SNU-NCC-61	18,19	14.2,15	18,19	8,9	17,21	X,Y	11,13	22,23
SNU-NCC-376	17,24	13	16	8,11	14,17	X,Y	10,13	19,21
SNU-NCC-377	17,20	13	16,17	8,11	17	X,Y	12	24

Supplementary Table 2. 15 genes associated with colorectal cancer

CELL LINE	*APC*			*KRAS*		
	nt	Nt change (a.a change)	Effects	nt	Nt change (a.a change)	Effects
SNU-1566	4364	c.4364delA (p.Asn1455fs)		38	c.38G>A (p.Gly13Asp)	Pathogenic
	5465	c.5465T>A (p.Val1822Asp)				
SNU-1983	4918	c.4918C>T (p.Arg1640Trp)		35	c.35G>A (p.Gly12Asp)	Pathogenic
	5465	c.5465T>A (p.Val1822Asp)				
SNU-2172	2442	c.2442delT (p.Phe814fs)		34	c.34G>A (p.Gly12Ser)	Pathogenic
	4452_4458	c.4452_4458del TGCTGAT (p.Asp1484fs)				
SNU-2297	5465	c.5465T>A (p.Val1822Asp)		Wt		
SNU-2303	673	c.673C>A (p.Leu225Ile)		Wt		
	4385	c.4385*>-AG (p.X1462X)	Pathogenic			

	5465	c.5465T>A (p.Val1822Asp)				
SNU-2353B	556	c.556delA (p.Arg186fs)		Wt		
	5465	c.5465T>A (p.Val1822Asp)				
SNU-2359	5465	c.5465T>A (p.Val1822Asp)		Wt		
SNU-2373B	2626	c.2626C>T (p.Arg876*)	Pathogenic	Wt		
	5465	c.5465T>A (p.Val1822Asp)				
SNU-2407	5465	c.5465T>A (p.Val1822Asp)		35	c.35G>A (p.Gly12Asp)	Pathogenic
	637	c.637C>T (p.Arg213*)	Pathogenic		c.38G>A (p.Gly13Asp)	
SNU-2423	4333	c.4333delA (p.Thr1445fs)		38		Pathogenic
	5465	c.5465T>A (p.Val1822Asp)				
SNU-2431	5465	c.5465T>A (p.Val1822Asp)		35	c.35G>T (p.Gly12Val)	Pathogenic
SNU-2465	2701	c.2701C>T (p.Gln901*)	Pathogenic	Wt		

	5465	c.5465T>A (p.Val1822Asp)				
SNU-2493	637	c.637C>T (p.Arg213*)	Pathogenic	Wt		
	5465	c.5465T>A (p.Val1822Asp)				
	1960	c.1960C>T (p.Gln654*)			c.35G>A (p.Gly12Asp)	
SNU-2536C	4271	c.4271delC (p.Pro1424fs)		35		pathogenic
	5465	c.5465T>A (p.Val1822Asp)				
	2626	c.2626C>T (p.Arg876*)	Pathogenic			
SNU-2621B	4666	c.4666dupA (p.Thr1556fs)	Pathogenic	Wt		
	5465	c.5465T>A (p.Val1822Asp)				
SNU-NCC-61	5465	c.5465T>A (p.Val1822Asp)		Wt		
SNU-NCC-81	5465	c.5465T>A (p.Val1822Asp)		Wt		
SNU-NCC-376	5465	c.5465T>A (p.Val1822Asp)		Wt		

SNU-NCC-377	1918	c.1918C>T (p.Arg640Trp)		35	c.35G>A (p.Gly12Asp)	Pathogenic
	607	c.607C>T (p.Arg203Trp)	Pathogenic			
	4348	c.4348C>T (p.Arg1450*)	Pathogenic			

CELL LINE	*BARF*			*PTEN*		
	nt	Nt change (a.a change)	Effects	nt	Nt change (a.a change)	Effects
SNU-1566	Wt			wt		
SNU-1983	Wt			491	c.491delA (p.Lys164fs)	Pathogenic
SNU-2172	Wt			Wt		
SNU-2297	Wt			Wt		
SNU-2303	Wt			Wt		
SNU-2353B	Wt			Wt		
SNU-2359	1799	c.1799T>A (p.Val600Glu)	pathogenic	Wt		
SNU-2373B	wt			Wt		
SNU-2407	wt			Wt		
SNU-2423	wt			Wt		

SNU-2431	wt			Wt		
SNU-2465	1799	c.1799T>A (p.Val600Glu)	pathogenic	Wt		
SNU-2493	wt			Wt		
SNU-2536C	wt			Wt		
SNU-2621B	wt			Wt		
SNU-NCC-61	1799	c.1799T>A (p.Val600Glu)	pathogenic	Wt		
SNU-NCC-81	Wt			Wt		
SNU-NCC-376	1799	c.1799T>A (p.Val600Glu)	pathogenic	wt		
SNU-NCC-377	wt			wt		

CELL LINE	*MLH1*			*MSH2*		
	nt	Nt change (a.a change)	Effects	nt	Nt change (a.a change)	Effects
SNU-1566	Wt			1220	c.1220T>G (p.Leu407Arg)	
SNU-1983	1997	c.1997G>A (p.Trp666*)	Pathogenic	2744	c.2744A>G (p.Gln915Arg)	
SNU-2172	Wt			2744	c.2744A>G (p.Gln915Arg)	
SNU-2297	Wt			2744	c.2744A>G	

					(p.Gln915Arg)
SNU-2303	998	c.998T>C (p.Leu333Pro)	Pathogenic	2744	c.2744A>G (p.Gln915Arg)
SNU-2353B	Wt			2744	c.2744A>G (p.Gln915Arg)
SNU-2359	655	c.655A>G (p.Ile219Val)		Wt	
SNU-2373B	Wt			Wt	
SNU-2407	Wt			2744	c.2744A>G (p.Gln915Arg)
SNU-2423	440	c.440G>A (p.Gly147Glu)		2744	c.2744A>G (p.Gln915Arg)
	655	c.655A>G (p.Ile219Val)			
	1721	c.1721T>C (p.Leu574Pro)	Pathogenic		
SNU-2431	Wt			Wt	
SNU-2465	Wt			2744	c.2744A>G (p.Gln915Arg)
SNU-2493	655	c.655A>G (p.Ile219Val)		Wt	
SNU-2536C	Wt			23	c.23C>T (p.Thr8Met)
				2744	c.2744A>G

					(p.Gln915Arg)	
SNU-2621B	Wt			2744	c.2744A>G (p.Gln915Arg)	
SNU-NCC-61	Wt			2744	c.2744A>G (p.Gln915Arg)	
SNU-NCC-81	Wt			2744	c.2744A>G (p.Gln915Arg)	
SNU-NCC-376	Wt			2744	c.2744A>G (p.Gln915Arg)	
SNU-NCC-377	Wt			2744	c.2744A>G (p.Gln915Arg)	

CELL LINE	*MSH6*			*PMS2*		
	nt	Nt change (a.a change)	Effects	nt	Nt change (a.a change)	Effects
SNU-1566	3261	c.3261delC (p.Phe1088fs)	Pathogenic	1532	c.1532C>T (p.Thr511Met)	
SNU-1983	116	c.116G>A (p.Gly39Glu)		Wt		
SNU-2172	116	c.116G>A (p.Gly39Glu)		Wt		
SNU-2297	Wt			1532	c.1532C>T (p.Thr511Met)	

SNU-2303	345	c.345*>-A (p.X115X)	Pathogenic	1303	c.1303A>G (p.Lys435Glu)
				1214	c.1214C>T (p.Thr405Met)
SNU-2353B	116	c.116G>A (p.Gly39Glu)		Wt	
SNU-2359	Wt			756	c.756T>G (p.Cys252Trp)
SNU-2373B	116	c.116G>A (p.Gly39Glu)		1408	c.1408C>T (p.Pro470Ser)
SNU-2407	Wt			1408	c.1408C>T (p.Pro470Ser)
SNU-2423	Wt			1621	c.1621A>G (p.Lys541Glu)
				1408	c.1408C>T (p.Pro470Ser)
SNU-2431	4068_4071	c.4068_4071dupGATT (p.Lys1358fs)		1621	c.1621A>G (p.Lys541Glu)
SNU-2465	Wt			1621	c.1621A>G (p.Lys541Glu)
SNU-2493	Wt			1621	c.1621A>G (p.Lys541Glu)
SNU-2536C	Wt			1621	c.1621A>G (p.Lys541Glu)

SNU-2621B	116	c.116G>A (p.Gly39Glu)		1621	c.1621A>G (p.Lys541Glu)	
	3261	c.3261delC (p.Phe1088fs)	Pathogenic	802	c.802delT (p.Tyr268fs)	
SNU-NCC-61	Wt			1621	c.1621A>G (p.Lys541Glu)	
SNU-NCC-81	Wt			1621	c.1621A>G (p.Lys541Glu)	
SNU-NCC-376	116	c.116G>A (p.Gly39Glu)		1621	c.1621A>G (p.Lys541Glu)	
				1408	c.1408C>T (p.Pro470Ser)	
SNU-NCC-377	116	c.116G>A (p.Gly39Glu)		1621	c.1621A>G (p.Lys541Glu)	
				1408	c.1408C>T (p.Pro470Ser)	

CELL LINE	*POLD1*			*POLE*		
	nt	Nt change (a.a change)	Effects	Nt	Nt change (a.a change)	Effects
SNU-1566	356	c.356G>A (p.Arg119His)		755	c.755C>T (p.Ala252Val)	

SNU-1983	1036	c.1036delG (p.Glu346fs)	6149_6151	c.6149_6151delTCT (p.Phe2050del)
SNU-2172	356	c.356G>A (p.Arg119His)	Wt	
SNU-2297	356	c.356G>A (p.Arg119His)	Wt	
SNU-2303	wt		674	c.674C>T (p.Ala225Val)
SNU-2353B	Wt		Wt	
SNU-2359	Wt		Wt	
SNU-2373B	Wt		Wt	
SNU-2407	Wt		Wt	
SNU-2423	356	c.356G>A (p.Arg119His)	Wt	
SNU-2431	Wt		Wt	
SNU-2465	Wt		755	c.755C>T (p.Ala252Val)
SNU-2493	Wt		Wt	
SNU-2536C	356	c.356G>A (p.Arg119His)	Wt	
SNU-2621B	665	c.665C>T (p.Pro222Leu)	Wt	
	2856	c.2856delG (p.Thr953fs)		

SNU-NCC-61	Wt			Wt		
SNU-NCC-81	356	c.356G>A (p.Arg119His)		WT		
SNU-NCC-376	wt			WT		
SNU-NCC-377	wt			WT		

Continued

CELL LINE	*SMAD4*			*STK11*		
	nt	Nt change (a.a change)	Effects	Nt	Nt change (a.a change)	Effects
SNU-1566	513	c.513G>C (p.Glu171Asp)		Wt		
SNU-1983	Wt			Wt		
SNU-2172	Wt			Wt		
SNU-2297	1157	c.1157G>A (p.Gly386Asp)	Pathogenic	Wt		
SNU-2303	Wt			Wt		
SNU-2353B	513	c.513G>C (p.Glu171Asp)		Wt		
SNU-2359	Wt			Wt		
SNU-2373B	Wt			Wt		
SNU-2407	Wt			Wt		
SNU-2423	513	c.513G>C		Wt		

		(p.Glu171Asp)				
SNU-2431	1324	c.1324C>T (p.Gln442*)	Pathogenic	Wt		
SNU-2465	513	c.513G>C (p.Glu171Asp)		Wt		
SNU-2493	513	c.513G>C (p.Glu171Asp)		Wt		
SNU-2536C	Wt			Wt		
SNU-2621B	Wt			487	c.487G>A (p.Gly163Ser)	pathogenic
SNU-NCC-61	513	c.513G>C (p.Glu171Asp)		Wt		
SNU-NCC-81	513	c.513G>C (p.Glu171Asp)		Wt		
SNU-NCC-376	Wt			Wt		
SNU-NCC-377	wt			Wt		

Continued

CELL LINE	*TP53*			*CDH1*		
	Nt	Nt change (a.a change)	Effects	Nt	Nt change (a.a change)	Effects
SNU-1566	215	c.215C>G (p.Pro72Arg)	Pathogenic	Wt		
SNU-1983	215	c.215C>G	Pathogenic	862	c.862G>T	

		(p.Pro72Arg)			(p.Asp288Tyr)
SNU-2172	437	c.437G>A (p.Trp146*)		Wt	
	215	c.215C>G (p.Pro72Arg)	Pathogenic		
SNU-2297	586	c.586C>T (p.Arg196*)	Pathogenic	Wt	
	215	c.215C>G (p.Pro72Arg)	Pathogenic		
SNU-2303	215	c.215C>G (p.Pro72Arg)	Pathogenic	Wt	
SNU-2353B	Wt			Wt	
SNU-2359	527	c.527G>T (p.Cys176Phe)	Pathogenic	Wt	
SNU-2373B	wt			Wt	
SNU-2407	440	c.440T>A (p.Val147Asp)		Wt	
	215	c.215C>G (p.Pro72Arg)	Pathogenic		
SNU-2423	215	c.215C>G (p.Pro72Arg)	Pathogenic	Wt	
SNU-2431	1009	c.1009C>T (p.Arg337Cys)	Pathogenic	Wt	
SNU-2465	1024	c.1024C>T	Pathogenic	Wt	

		(p.Arg342*)				
SNU-2493	734	c.734G>A (p.Gly245Asp)	Pathogenic	Wt		
	215	c.215C>G (p.Pro72Arg)	Pathogenic			
SNU-2536C	318	c.318C>G (p.Ser106Arg)		Wt		
	215	c.215C>G (p.Pro72Arg)	Pathogenic			
SNU-2621B	215	c.215C>G (p.Pro72Arg)	Pathogenic	220	c.220C>T (p.Arg74*)	Pathogenic
				2398	c.2398delC (p.Arg800fs)	Pathogenic
SNU-NCC-61	980,981	c.980_981dupAT (p.Phe328fs)		Wt		
	31	c.31G>C (p.Glu11Gln)				
SNU-NCC-81	215	c.215C>G (p.Pro72Arg)	Pathogenic	Wt		
SNU-NCC-376	636	c.636delT (p.Arg213fs)	Pathogenic	Wt		
	215	c.215C>G (p.Pro72Arg)	Pathogenic			
SNU-NCC-377	376	c.376-1G>A	Pathogenic	Wt		

CELL LINE	*EPCAM*		
	nt	Nt change (a.a change)	Effects
SNU-1566	Wt		
SNU-1983	211	c.211dupG (p.Val71fs)	
	428	c.428T>C (p.Met143Thr)	
SNU-2172	428	c.428T>C (p.Met143Thr)	
SNU-2297	428	c.428T>C (p.Met143Thr)	
SNU-2303	Wt		
SNU-2353B	211	c.211dupG (p.Val71fs)	
SNU-2359	211	c.211dupG (p.Val71fs)	
	428	c.428T>C (p.Met143Thr)	
SNU-2373B	211	c.211dupG	

		(p.Val71fs)
	428	c.428T>C (p.Met143Thr)
SNU-2407	428	c.428T>C (p.Met143Thr)
	211	c.211dupG (p.Val71fs)
SNU-2423	428	c.428T>C (p.Met143Thr)
	599	c.599C>T (p.Thr200Met)
SNU-2431	428	c.428T>C (p.Met143Thr)
SNU-2465	428	c.428T>C (p.Met143Thr)
SNU-2493	Wt	
SNU-2536C	211	c.211dupG (p.Val71fs)
	428	c.428T>C (p.Met143Thr)
SNU-2621B	428	c.428T>C (p.Met143Thr)
SNU-NCC-61	211	c.211dupG (p.Val71fs)

	428	c.428T>C (p.Met143Thr)
	599	c.599C>T (p.Thr200Met)
SNU-NCC-81	428	c.428T>C (p.Met143Thr)
	344	c.344T>C (p.Met115Thr)
SNU-NCC-376	428	c.428T>C (p.Met143Thr)
SNU-NCC-377	428	c.428T>C (p.Met143Thr)
